# Supplementary material for: The walking speed-dependency of gait variability in bilateral vestibulopathy and its association with clinical tests of vestibular function
Source: Sci Rep. 2019 Dec 5;9:18392. doi: 10.1038/s41598-019-54605-0 (PMC6895118; doi:10.1038/s41598-019-54605-0)
Supplement: Supplementary file 1 — Supplement [file 41598_2019_54605_MOESM1_ESM.pdf]

Supplementary Information for

The walking speed-dependency of gait variability in bilateral vestibulopathy and its association with clinical tests of vestibular function

Scientific Reports

Christopher McCrum[1,2]*, Florence Lucieer[3], Raymond van de Berg[3,4], Paul Willems[1], Angélica Pérez Fornos[5], Nils Guinand[5], Kiros Karamanidis[6], Herman Kingma[3,4], Kenneth Meijer[1]

[1]Department of Nutrition and Movement Sciences, NUTRIM School of Nutrition and Translational Research in Metabolism, Maastricht University Medical Centre+, Maastricht, The Netherlands

[2]Institute of Movement and Sport Gerontology, German Sport University Cologne, Germany

[3]Division of Balance Disorders, Department of Otolaryngology, Head and Neck Surgery, Maastricht University Medical Centre+, Maastricht, The Netherlands

[4]Faculty of Physics, Tomsk State University, Tomsk, Russian Federation

[5]Service of Otorhinolaryngology and Head and Neck Surgery, Department of Clinical Neurosciences, Geneva University Hospitals, Geneva, Switzerland

[6]Sport and Exercise Science Research Centre, School of Applied Sciences, London South Bank University, London, UK

*Correspondence:
Christopher McCrum
Department of Nutrition and Movement Sciences
Maastricht University,
PO Box 616, Maastricht, 6200 MD, The Netherlands
Phone: +31433881621
Email: chris.mccrum@maastrichtuniversity.nl

Supplementary Table 1: Bonferroni pairwise between group comparison results for the means of the gait parameters

Walking Speed (m/s)	Groups	Step Time Means			Step Length Means			Double Support Time Means			Step Width Means		
		**Mean Diff.**	**95.00% CI of diff.**	**Adjusted P Value**	**Mean Diff.**	**95.00% CI of diff.**	**Adjusted P Value**	**Mean Diff.**	**95.00% CI of diff.**	**Adjusted P Value**	**Mean Diff.**	**95.00% CI of diff.**	**Adjusted P Value**
0.4	BVP vs. Young	-0.1579	-0.2419 to -0.07384	**0.0001**	-0.06307	-0.09654 to -0.02960	**0.0001**	-0.07791	-0.1170 to -0.03885	**<0.0001**	0.03707	0.003385 to 0.07075	**0.0268**
	BVP vs. Older	-0.09576	-0.1938 to 0.002244	0.057	-0.03835	-0.07742 to 0.0007265	0.0556	-0.02706	-0.07522 to 0.02110	0.4786	0.03175	-0.009976 to 0.07347	0.1867
	Young vs. Older	0.06212	-0.04178 to 0.1660	0.4046	0.02472	-0.01663 to 0.06607	0.4044	0.05086	0.0008404 to 0.1009	**0.0454**	-0.00532	-0.04651 to 0.03587	>0.9999
0.6	BVP vs. Young	-0.09504	-0.1465 to -0.04361	**0.0002**	-0.05714	-0.08806 to -0.02622	**0.0002**	-0.03592	-0.05997 to -0.01188	**0.0024**	0.02883	-0.006725 to 0.06439	0.1482
	BVP vs. Older	-0.05925	-0.1277 to 0.009149	0.1047	-0.03606	-0.07720 to 0.005079	0.099	-0.01187	-0.03891 to 0.01517	0.7921	0.03253	-0.01095 to 0.07602	0.2005
	Young vs. Older	0.03579	-0.03479 to 0.1064	0.5979	0.02108	-0.02143 to 0.06358	0.627	0.02405	-0.006358 to 0.05446	0.1572	0.003702	-0.03852 to 0.04592	>0.9999
0.8	BVP vs. Young	-0.05854	-0.09343 to -0.02365	**0.0004**	-0.04711	-0.07525 to -0.01898	**0.0005**	-0.01993	-0.03893 to -0.0009376	**0.0374**	0.01668	-0.02021 to 0.05358	0.7954
	BVP vs. Older	-0.0398	-0.08653 to 0.006931	0.1149	-0.03217	-0.06972 to 0.005381	0.1118	-0.009608	-0.02740 to 0.008184	0.5388	0.02243	-0.01548 to 0.06034	0.4394
	Young vs. Older	0.01874	-0.02655 to 0.06403	0.8688	0.01494	-0.02150 to 0.05138	0.8821	0.01033	-0.01040 to 0.03105	0.6294	0.005752	-0.03036 to 0.04187	>0.9999
1	BVP vs. Young	-0.05273	-0.08212 to -0.02334	**0.0002**	-0.05325	-0.08276 to -0.02374	**0.0002**	-0.0124	-0.02665 to 0.001852	0.1062	0.01449	-0.02451 to 0.05349	>0.9999
	BVP vs. Older	-0.02875	-0.06112 to 0.003616	0.0959	-0.02911	-0.06122 to 0.002991	0.0864	-0.00682	-0.02207 to 0.008430	0.7972	0.0166	-0.02325 to 0.05645	0.9078
	Young vs. Older	0.02398	-0.006335 to 0.05429	0.1569	0.02413	-0.006074 to 0.05434	0.1507	0.005578	-0.009967 to 0.02112	>0.9999	0.002111	-0.03669 to 0.04091	>0.9999
1.2	BVP vs. Young	-0.03527	-0.05426 to -0.01627	**0.0002**	-0.04224	-0.06470 to -0.01978	**0.0001**	-0.004739	-0.01834 to 0.008861	>0.9999	0.01157	-0.02591 to 0.04906	>0.9999
	BVP vs. Older	-0.01281	-0.03491 to 0.009291	0.4441	-0.01505	-0.04133 to 0.01123	0.4584	-0.001326	-0.01452 to 0.01187	>0.9999	0.01897	-0.02107 to 0.05900	0.7251
	Young vs. Older	0.02246	-0.0008739 to 0.04579	0.0618	0.02719	-0.0004576 to 0.05483	0.0549	0.003413	-0.01246 to 0.01929	>0.9999	0.007391	-0.03035 to 0.04514	>0.9999
1.4	BVP vs. Young	-0.02955	-0.04798 to -0.01112	**0.001**	-0.04111	-0.06684 to -0.01537	**0.001**	-0.002945	-0.01446 to 0.008574	>0.9999	0.01293	-0.02312 to 0.04897	>0.9999
	BVP vs. Older	-0.009134	-0.02823 to 0.009961	0.7002	-0.01253	-0.03915 to 0.01409	0.7227	-0.003735	-0.01521 to 0.007745	>0.9999	0.009436	-0.03317 to 0.05204	>0.9999
	Young vs. Older	0.02042	0.0001919 to 0.04065	**0.0474**	0.02858	0.0003932 to 0.05676	**0.0461**	-0.00079	-0.01374 to 0.01216	>0.9999	-0.00349	-0.04146 to 0.03448	>0.9999
1.6	BVP vs. Young	-0.01865	-0.03602 to -0.001278	**0.0324**	-0.02908	-0.05752 to -0.0006455	**0.0438**	-0.001623	-0.01276 to 0.009513	>0.9999	0.005952	-0.03517 to 0.04707	>0.9999
	BVP vs. Older	-0.006196	-0.02836 to 0.01597	>0.9999	-0.01025	-0.04572 to 0.02522	>0.9999	-0.002359	-0.01579 to 0.01108	>0.9999	0.002257	-0.03830 to 0.04282	>0.9999
	Young vs. Older	0.01245	-0.01100 to 0.03591	0.5373	0.01883	-0.01903 to 0.05669	0.6219	-0.000736	-0.01479 to 0.01332	>0.9999	-0.003695	-0.03929 to 0.03190	>0.9999

Supplementary Table 2: Bonferroni pairwise between group comparison results for the coefficients of variation of the gait parameters

Walking Speed (m/s)	Groups	Step Time CV			Step Length CV			Double Support Time CV			Step Width CV		
		**Mean Diff.**	**95.00% CI of diff.**	**Adjusted P Value**	**Mean Diff.**	**95.00% CI of diff.**	**Adjusted P Value**	**Mean Diff.**	**95.00% CI of diff.**	**Adjusted P Value**	**Mean Diff.**	**95.00% CI of diff.**	**Adjusted P Value**
0.4	BVP vs. Young	1.271	-0.09019 to 2.631	0.073	2.791	0.8092 to 4.773	**0.004**	4.523	1.343 to 7.704	**0.0033**	0.7604	-2.313 to 3.834	>0.9999
	BVP vs. Older	0.3193	-1.749 to 2.388	>0.9999	1.611	-1.029 to 4.252	0.3731	1.269	-4.099 to 6.637	>0.9999	1.46	-3.380 to 6.299	>0.9999
	Young vs. Older	-0.9513	-3.125 to 1.222	0.79	-1.18	-3.990 to 1.630	0.8556	-3.254	-8.710 to 2.201	0.3927	0.6991	-4.033 to 5.432	>0.9999
0.6	BVP vs. Young	1.355	0.4934 to 2.216	**0.0009**	2.699	1.153 to 4.246	**0.0004**	3.953	1.783 to 6.123	**0.0001**	4.984	0.9730 to 8.994	**0.0102**
	BVP vs. Older	0.9247	-0.5926 to 2.442	0.3752	1.704	-0.3164 to 3.724	0.1182	2.94	-1.040 to 6.920	0.1989	4.169	-1.194 to 9.531	0.1745
	Young vs. Older	-0.4301	-1.909 to 1.049	>0.9999	-0.9957	-3.101 to 1.109	0.691	-1.013	-4.941 to 2.915	>0.9999	-0.8149	-5.417 to 3.787	>0.9999
0.8	BVP vs. Young	1.201	0.5513 to 1.850	**<0.0001**	2.606	1.459 to 3.754	**<0.0001**	3.473	1.361 to 5.586	**0.0006**	10.67	4.704 to 16.64	**0.0002**
	BVP vs. Older	0.5263	-0.6012 to 1.654	0.7134	1.15	-0.3300 to 2.630	0.1724	2.72	0.03094 to 5.410	**0.0468**	6.319	-3.386 to 16.02	0.3216
	Young vs. Older	-0.6742	-1.725 to 0.3768	0.3065	-1.456	-2.859 to -0.05344	**0.0404**	-0.7528	-3.407 to 1.901	>0.9999	-4.355	-12.93 to 4.216	0.5519
1	BVP vs. Young	0.8551	0.01044 to 1.700	**0.0466**	2.091	0.9474 to 3.234	**0.0001**	3.203	0.5901 to 5.815	**0.012**	12.86	6.892 to 18.83	**<0.0001**
	BVP vs. Older	0.2881	-0.5151 to 1.091	>0.9999	0.8791	-0.4269 to 2.185	0.2971	1.236	-1.258 to 3.731	0.6655	11.18	4.064 to 18.29	**0.001**
	Young vs. Older	-0.567	-1.513 to 0.3790	0.4038	-1.212	-2.416 to -0.007390	**0.0483**	-1.966	-4.525 to 0.5927	0.177	-1.681	-6.651 to 3.289	>0.9999
1.2	BVP vs. Young	0.6428	0.1572 to 1.128	**0.0058**	1.579	0.7094 to 2.448	**0.0002**	2.907	0.8770 to 4.937	**0.0027**	14.37	8.318 to 20.42	**<0.0001**
	BVP vs. Older	0.2557	-0.3420 to 0.8533	0.8616	0.7506	-0.01438 to 1.515	0.056	1.651	-0.1934 to 3.495	0.0932	11.22	3.168 to 19.27	**0.0038**
	Young vs. Older	-0.3872	-0.9284 to 0.1541	0.2271	-0.8281	-1.647 to -0.009037	**0.047**	-1.256	-2.748 to 0.2368	0.1194	-3.15	-9.550 to 3.250	0.6027
1.4	BVP vs. Young	0.7897	0.3740 to 1.205	**<0.0001**	1.492	0.7862 to 2.198	**<0.0001**	2.492	0.1591 to 4.824	**0.0329**	15.22	7.806 to 22.63	**<0.0001**
	BVP vs. Older	0.4487	-0.02622 to 0.9237	0.0695	0.6927	0.01951 to 1.366	**0.0419**	1.558	-0.6551 to 3.771	0.2584	13.38	4.020 to 22.74	**0.0029**
	Young vs. Older	-0.341	-0.7297 to 0.04760	0.0983	-0.7993	-1.422 to -0.1769	**0.0092**	-0.9334	-3.036 to 1.170	0.7864	-1.842	-9.031 to 5.347	>0.9999
1.6	BVP vs. Young	0.4406	0.06548 to 0.8158	**0.0169**	0.923	0.2817 to 1.564	**0.0029**	2.356	0.3356 to 4.376	**0.0178**	13.11	5.274 to 20.95	**0.0005**
	BVP vs. Older	0.1737	-0.2706 to 0.6179	0.9734	0.3466	-0.2665 to 0.9596	0.4899	1.059	-1.125 to 3.243	0.6811	14.73	7.518 to 21.94	**<0.0001**
	Young vs. Older	-0.267	-0.7029 to 0.1690	0.3732	-0.5765	-1.174 to 0.02123	0.0611	-1.297	-3.418 to 0.8240	0.3782	1.619	-3.208 to 6.447	>0.9999

Supplementary Table 3: Bonferroni pairwise between BVP All Gait vs. BVP Part Gait comparison results for the coefficients of variation of the gait parameters

Walking Speed (m/s)	Step Time CV			Step Length CV			Double Support Time CV			Step Width CV		
	Mean Diff.	95.00% CI of diff.	Adjusted P Value	Mean Diff.	95.00% CI of diff.	Adjusted P Value	Mean Diff.	95.00% CI of diff.	Adjusted P Value	Mean Diff.	95.00% CI of diff.	Adjusted P Value
0.4	-1.572	-3.820 to 0.6765	0.3059	-3.163	-6.032 to -0.2933	**0.0259**	-4.748	-11.28 to 1.787	0.2531	-0.4195	-6.080 to 5.241	>0.9999
0.6	-1.259	-3.216 to 0.6981	0.4225	-2.432	-5.321 to 0.4568	0.1305	-2.8	-7.624 to 2.024	0.5944	-1.423	-11.73 to 8.881	>0.9999
0.8	-1.241	-2.925 to 0.4423	0.2441	-1.864	-4.438 to 0.7090	0.2652	-3.054	-7.209 to 1.100	0.2551	1.35	-12.73 to 15.42	>0.9999
1	-0.7285	-2.039 to 0.5822	0.684	-1.316	-4.399 to 1.768	>0.9999	-3.068	-9.497 to 3.361	0.9497	-1.021	-16.51 to 14.47	>0.9999
1.2	-0.3443	-1.341 to 0.6527	>0.9999	-0.202	-1.677 to 1.273	>0.9999	-1.29	-5.509 to 2.930	>0.9999	-6.166	-23.90 to 11.57	>0.9999
1.4	-1.089	-2.639 to 0.4610	0.212	-1.134	-3.709 to 1.440	0.8482	-5.092	-15.66 to 5.471	0.6228	-8.85	-39.64 to 21.94	>0.9999
